# Standardised imaging pipeline for phenotyping mouse laterality defects and associated heart malformations, at multiple scales and multiple stages

**DOI:** 10.1242/dmm.038356

**Published:** 2019-07-09

**Authors:** Audrey Desgrange, Johanna Lokmer, Carmen Marchiol, Lucile Houyel, Sigolène M. Meilhac

**Affiliations:** 1 Imagine-Institut Pasteur, Laboratory of Heart Morphogenesis, 75015 Paris, France; 2 INSERM UMR1163, 75015 Paris, France; 3 Université Paris Descartes, Sorbonne Paris-Cité, 75006 Paris, France; 4 INSERM U1016, Institut Cochin, 75014 Paris, France; 5 CNRS UMR8104, 75014 Paris, France; 6 Unité de Cardiologie Pédiatrique et Congénitale, Hôpital Necker Enfants Malades, Centre de référence des Malformations Cardiaques Congénitales Complexes-M3C, APHP, 75015 Paris, France

**Keywords:** Laterality defects, Heterotaxy, Congenital heart defects, *Situs*, 3D imaging, Left-right asymmetry

## Abstract

Laterality defects are developmental disorders resulting from aberrant left/right patterning. In the most severe cases, such as in heterotaxy, they are associated with complex malformations of the heart. Advances in understanding the underlying physiopathological mechanisms have been hindered by the lack of a standardised and exhaustive procedure in mouse models for phenotyping left/right asymmetries of all visceral organs. Here, we have developed a multimodality imaging pipeline, which combines non-invasive micro-ultrasound imaging, micro-computed tomography (micro-CT) and high-resolution episcopic microscopy (HREM) to acquire 3D images at multiple stages of development and at multiple scales. On the basis of the position in the uterine horns, we track in a single individual, the progression of organ asymmetry, the *situs* of all visceral organs in the thoracic or abdominal environment, and the fine anatomical left/right asymmetries of cardiac segments. We provide reference anatomical images and organ reconstructions in the mouse, and discuss differences with humans. This standardised pipeline, which we validated in a mouse model of heterotaxy, offers a fast and easy-to-implement framework. The extensive 3D phenotyping of organ asymmetry in the mouse uses the clinical nomenclature for direct comparison with patient phenotypes. It is compatible with automated and quantitative image analyses, which is essential to compare mutant phenotypes with incomplete penetrance and to gain mechanistic insight into laterality defects.

## Abstract (precis)

**Summary:** Laterality defects, which combine anomalies in several visceral organs, are challenging to phenotype. We have developed here a standardised approach for multimodality 3D imaging in mice, generating quantifiable phenotypes.

## INTRODUCTION

Laterality defects are developmental disorders caused by impaired left-right patterning. Collectively, these disorders affect up to 1 in 2000 live births and comprise a spectrum of malformations ranging from asymptomatic *situs inversus* to severe heterotaxy (Desgrange et al., 2018; Lin et al., 2014). Heterotaxy corresponds to abnormal symmetry (isomerism) of, and/or *situs* discordance between, visceral organs (Van Praagh, 2006). The phenotype is variable: many visceral organs (heart, lung, spleen, stomach, intestine, liver and pancreas) can be targeted and functionally impaired, with an abnormal position in the abdominal or thoracic cavity (*situs*) or an impaired asymmetric shape. Diagnosis is made on the combination of three out of eight criteria, including abdominal *situs* abnormality, spleen abnormality, isomerism of bronchi and the lungs, biliary atresia, intestinal malrotation, congenital heart defects, isomerism of the atrial appendages and systemic venous anomalies (Lin et al., 2014). The prognosis of heterotaxy mainly depends on the cardiac malformation, which can be complex with profound functional effects, such as abnormal oxygen supply or obstructed blood flow. Diagnosis of complex congenital heart defects is performed using the segmental approach developed by Van Praagh (1972), analysing separately the atria, the ventricles and the great arteries. Anatomical features are used to differentiate each cardiac segment (left/right ventricle, Van Praagh and Van Praagh, 1972; left/right atrium, Uemura et al., 1995), whereas the heart phenotype is based on the position in the thoracic cavity of the morphological left and right cardiac segments and their connection relative to each other (Jacobs et al., 2007).

In the past two decades, experiments in the mouse/animal model have provided insight into how left-right patterning is established in the early embryo (Hamada and Tam, 2014) by the activity of a left-right organiser, in which fluid flow generated by motile cilia has a central role (Nonaka et al., 1998). Heart looping at embryonic day (E) 8.5 is the first morphological sign of left/right asymmetric morphogenesis (Desgrange et al., 2018). During this process, the heart tube, which is initially straight with the right ventricle lying cranially to the left ventricle, becomes helical. This process establishes the relative position of the different cardiac segments, such that the right ventricle lies on the right side of the left ventricle (Le Garrec et al., 2017). Thus, the definitive left/right position of organ segments is the result of asymmetric morphogenesis. By contrast, gastrulation marks a physical separation, on either side of the primitive streak, between left and right precursor cells; cells receive asymmetric signalling, as for example from the left determinant Nodal (Collignon et al., 1996). Left and right precursor cells can be traced to assess how they contribute to different regions of an organ. Thus, DiI (1,1′-dioctadecyl-3,3,3′,3′-tetramethylindo-carbocyanine perchlorate) labelling and clonal analyses have provided insight into the left/right embryological origin of liver lobes (Weiss et al., 2016) and cardiac segments (Domínguez et al., 2012; Lescroart et al., 2010, 2012). The mechanisms of asymmetric organ morphogenesis remain largely unknown, however.

The mouse provides a good model for the study of laterality defects, given the array of genetics tools available to reproduce genetic alterations and considering the anatomical similarities between mammals. There are anatomical variations between the mouse and human, however, which can be extracted from fragmented analyses of individual mouse organs: the lungs (Thiesse et al., 2010), liver (Fiebig et al., 2012), gastrointestinal tract (Freeling and Rezvani, 2016; Nguyen et al., 2015), cardiac veins (Ciszek et al., 2007; Kaufman and Richardson, 2005) and heart (Webb et al., 1996). Thus, a comprehensive description of laterality features in all mouse visceral organs, and its relevance to clinical diagnosis, has been lacking. Analyses of mouse mutant lines have been limited in several respects. Mutations of genes involved in the left-right organiser lead to several categories of phenotype, which are not fully penetrant and often observed with a randomised frequency. This is the case for mutations impairing ciliogenesis, which are associated with randomised heart looping direction in the embryo and congenital heart defects at birth (Layton, 1976), as exemplified by *Rpgrip1l* mutants (Vierkotten et al., 2007). Phenotype randomisation requires observations in a high number of individuals and hinders correlation of phenotypes observed in different individuals at different stages. The phenotype description is often incomplete, focusing on a few organs of interest and with quantification in a small sample size. Phenotypes can be described in invasive open chest dissections or explanted organs, thus limiting conclusions on organ *situs*. Alternatively, complex malformations can be diagnosed in 2D histological sections, but these are associated with tissue distortion and might miss important features without the third dimension and the continuity of structures. In addition, phenotypes described by developmental biologists do not always refer to the clinical nomenclature, hence limiting the possibility of cross-correlations with patient phenotypes. Thus, understanding the origin of laterality defects has been hindered by the lack of a standardised and exhaustive procedure for phenotyping. Advances in 3D volumetric imaging have provided the possibility to image the non-transparent mouse organism at a smaller scale compared with humans. *In utero* mouse development is now accessible by high-frequency micro-ultrasound imaging (Foster et al., 2011) or optical coherence tomography (OCT) (Syed et al., 2011). With other approaches, such as optical projection tomography (OPT) (Sharpe et al., 2002), OCT (Lopez et al., 2015) or X-ray micro-computed tomography (micro-CT) (Degenhardt et al., 2010), the structure of internal organs can be reconstructed. With a higher imaging depth and wider field of view, micro-CT was selected for routine screening of mouse mutants (Wong et al., 2014). Finally, histological based approaches, such as episcopic fluorescence image capture (EFIC) and high-resolution episcopic microscopy (HREM) (Mohun and Weninger, 2012), reach very high spatial resolution and are able to resolve subtle anatomical variations, as exemplified in the heart (Geyer et al., 2017). To fully describe laterality defects, it is a challenge to combine images at multiple scales to resolve the *situs* of all visceral organs together with fine anatomical left/right features; it is also necessary to combine images at multiple stages of development to understand the origin of the defects.

Here, we report a novel multimodality imaging pipeline to phenotype laterality defects in 3D in the mouse. To assess the shape of the embryonic heart loop *in vivo*, we first perform non-invasive micro-ultrasound imaging on a pregnant mouse. By recording the position of each embryo in the uterine horns, correlations with another stage of development is possible. Each developing mouse is tracked just before birth, at E18.5, by micro-CT to determine the *situs* of visceral organs in their endogenous environment (i.e. without dissection), and to resolve vascular connections. Finally, to evaluate the fine cardiac anatomy, including left/right features, we acquired images of the isolated E18.5 heart by HREM. Based on sequential established imaging modalities, the novel imaging pipeline is a standardised procedure for the extensive phenotyping of organ laterality in the mouse. To guide phenotyping, we provide state-of-the-art reference images annotated with the clinical nomenclature.

## RESULTS

### Imaging the embryonic heart loop *in utero* by micro-ultrasound imaging

**Fig. 1.**
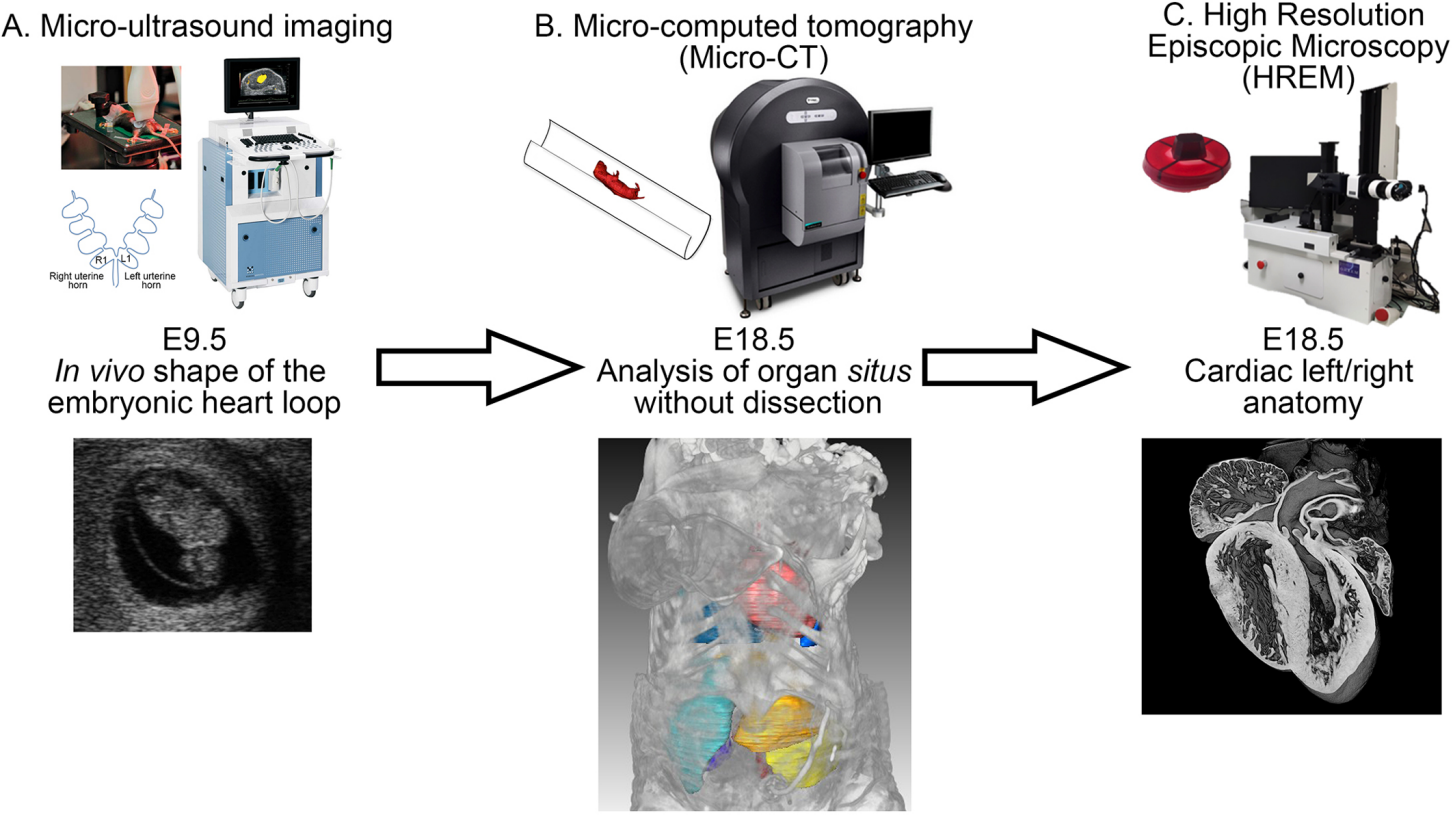
**Multimodality imaging pipeline of left/right asymmetries in the mouse.** (A) Micro-ultrasound imaging of a pregnant mouse to assess *in vivo* the shape of the embryonic heart loop at E9.5. The position of the embryo in the uterine horn is recorded as shown schematically, L1 and R1 being the first embryo next to the vagina in the left and right horns, respectively. (B) Micro-CT of the same individuals at E18.5, to image the *situs* of thoracic and abdominal organs. (C) HREM on the explanted E18.5 heart to image its left/right anatomical features. In each panel, the sample preparation is shown on the top left, the equipment on the top right and an example of an image is shown below: (A) a micro-ultrasound snapshot; (B) a 3D projection with segmented organ contours; and (C) a 3D projection.

**Fig. 2.**
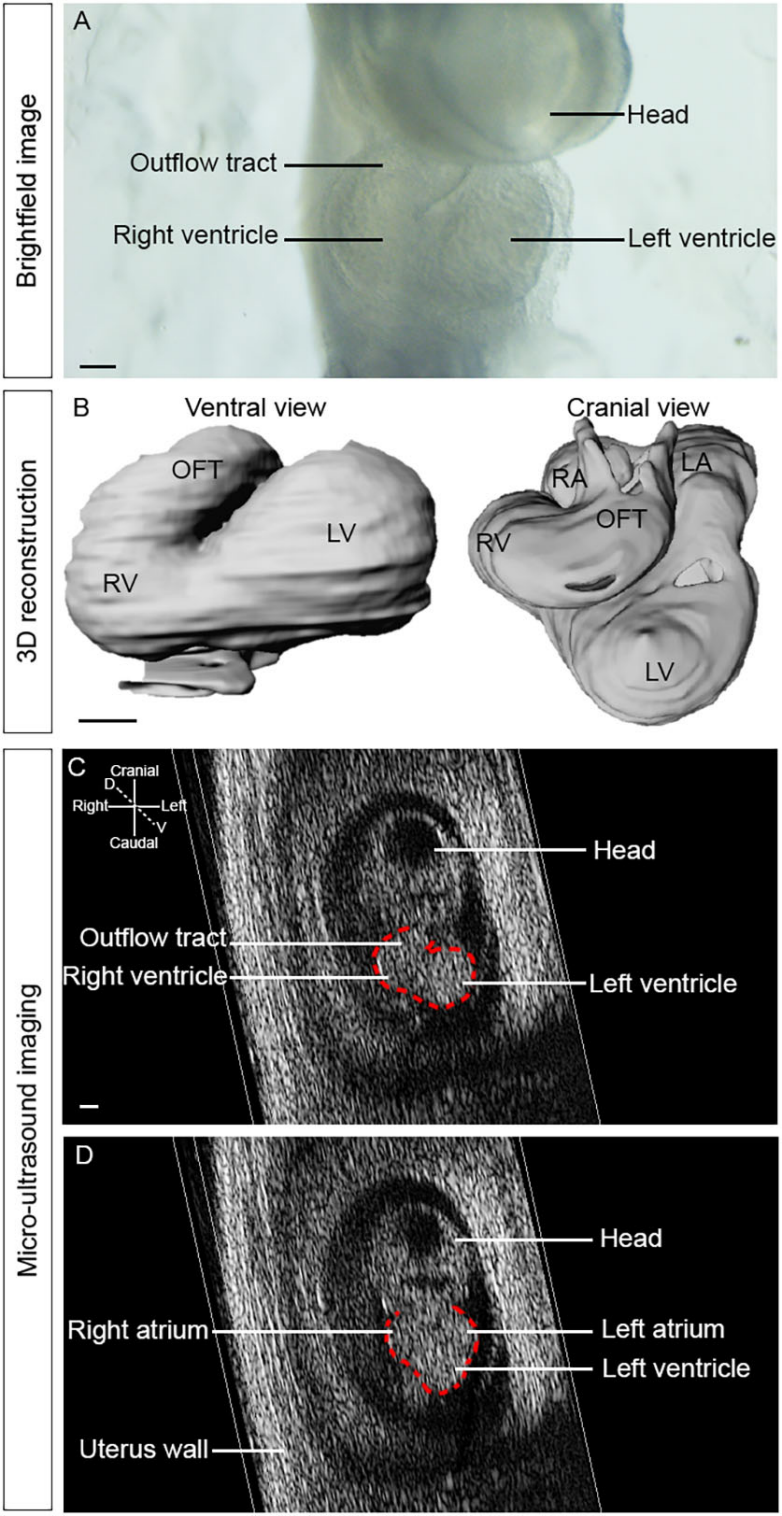
**Micro-ultrasound imaging of the embryonic heart loop *in utero*.** (A) Brightfield image of an explanted embryo at E9.5, showing the shape of the embryonic heart. (B) A 3D reconstruction on a ventral and cranial view, after segmentation of HREM images at E9.5, showing the helical shape of the embryonic heart. (C) Ventral and (D) dorsal sections of a 3D+t image of an E9.5 wild-type embryo acquired by micro-ultrasound imaging *in utero*. The contour of the embryonic heart is outlined in red. D, dorsal; LA, left atrium; LV, left ventricle; OFT, outflow tract; RA, right atrium; RV, right ventricle; V, ventral. Scale bars: 100 µm.

Heart looping is the first morphological sign of left/right asymmetry in the developing embryo and anomalies in this process are associated with congenital heart defects (see Desgrange et al., 2018). To be able to correlate the shape of the heart at two different stages, imaging within the same individual is required. As the first step of an imaging pipeline (Fig. 1), we used non-invasive micro-ultrasound imaging to evaluate the shape of the embryonic heart loop, without perturbing embryo development. We selected E9.5 as a stage when heart looping is complete (Fig. 2A), and used 3D reconstruction of heart shape in fixed samples (Fig. 2B, Movie 1) as a framework of analysis for lower resolution images. We show that in a pregnant mouse we can identify individual embryos, each within a deciduum, by micro-ultrasound imaging. Each embryo is numbered according to its position in the uterine horns (Fig. 1A). At necropsy 9 days after imaging, the number of E18.5 foetuses found in each uterine horn was consistent (6 and 3 in litter #1, 3 and 5 in litter #2, 4 and 3 in litter #3, 4 and 4 in litter #4, on the left and right, respectively). A total of 31/32 foetuses were collected alive, in accordance with the good viability of embryos after micro-ultrasound imaging. For a standardised analysis of the embryonic heart shape, a projection of the 3D micro-ultrasound images was generated and coronal tissue sections extracted, independently of the orientation of image acquisition. In coronal sections, we used the head and tail as landmarks of the cranio-caudal axis of the embryo, and the heart and neural tube for its ventral-dorsal axis (Movie 2). On the basis of these two axes, the orientation of the left/right axis of the embryo can be determined (Fig. 2C,D). At E9.5, the fast heartbeat facilitates the localisation of the heart tube (Movie 2). The analysis of the shape of the embryonic heart is based on its organisation in distinct regions, positioned sequentially along the axis of the cardiac tube (Le Garrec et al., 2017). The outflow tract can be identified as the connection of the heart tube to the cranio-dorsal part of the embryo, whereas the right and left ventricles follow ventrally, separated by a sulcus, on the right and left of wild-type embryos, respectively (Fig. 2C). The atria are more dorsal and caudal and can be seen as two chambers on the right and left of wild-type embryos (Fig. 2D). Thus, we show that micro-ultrasound imaging is an appropriate and non-invasive method to assess the overall shape of the embryonic heart *in vivo*, as early as E9.5.

### Imaging the *situs* of thoracic and abdominal organs in foetuses by micro-CT

To assess the *situs* of all visceral organs, which can potentially be abnormal in laterality defects, a rapid imaging procedure to resolve several organs inside the body was required. Micro-CT, with a field of 10×10×10 mm, can acquire images of all thoracic and abdominal organs within 3 min, without any dissection of the foetus or neonate mouse. To be able to correlate phenotypes at birth with images of the embryo, samples were collected at E18.5, just before birth, when their position in the uterus can be tracked. Micro-CT provides 3D images, which can be segmented to reconstruct organ shapes in 3D or can be resectioned optically in any relevant orientation.

**Fig. 3.**
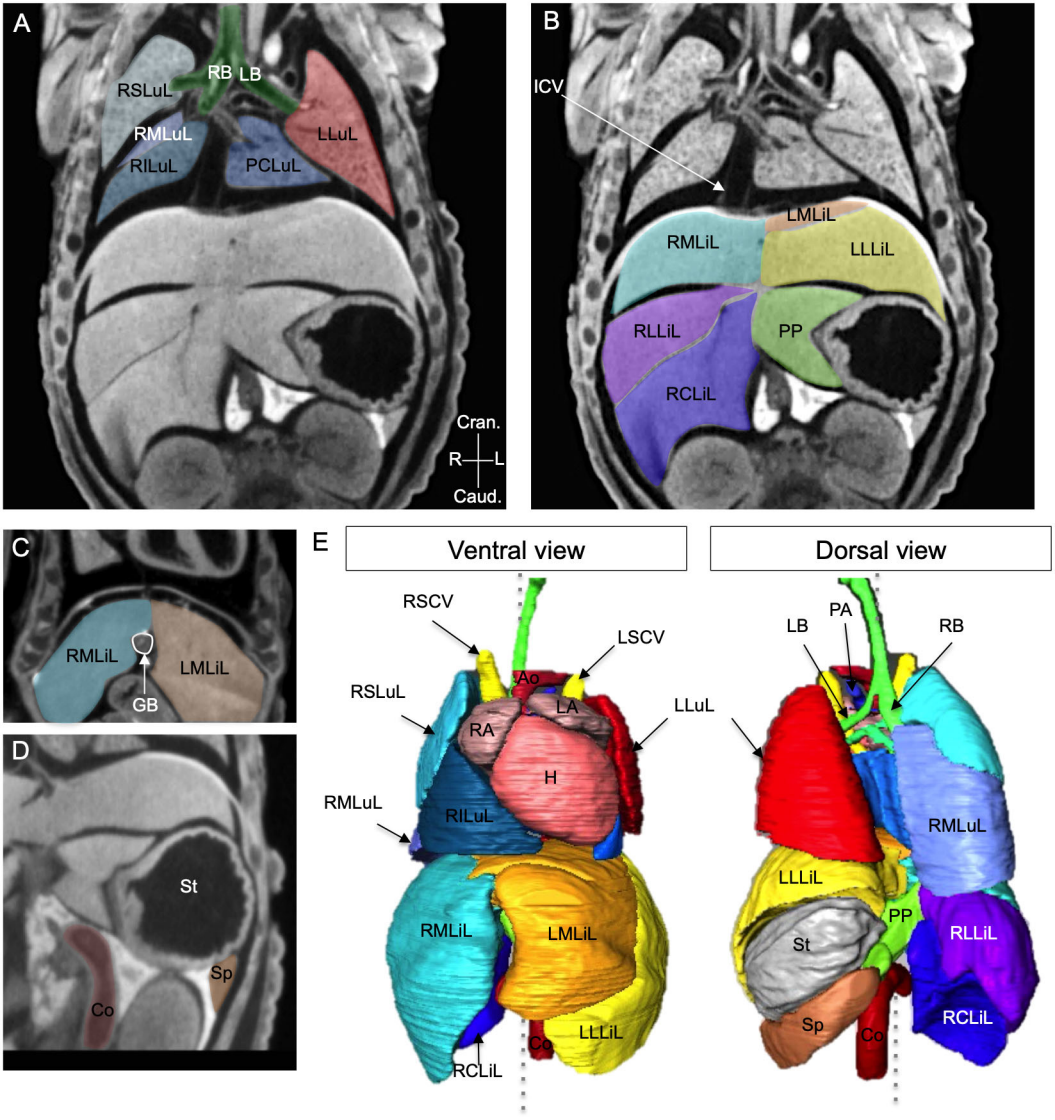
**Imaging of the *situs* of thoracic and abdominal organs by micro-CT at E18.5.** (A-D) Coronal sections from 3D images acquired by micro-CT of wild-type foetuses at E18.5, showing the thoracic and abdominal organs highlighted in different colours. (E) 3D reconstruction of the shape and position of organs within the body, from a ventral or dorsal view. The grey dotted line represents the plane of bilateral symmetry. Ao, aorta; Co, colon; Cran., cranial; Caud., caudal; GB, gall bladder; H, heart; ICV, inferior caval vein; L, left; LA, left atrium; LB, left bronchus; LLLiL, left lateral liver lobe; LLuL, left lung lobe; LMLiL, left medial liver lobe; LSCV, left superior caval vein; PA, pulmonary artery; PCLuL, post-caval (right) lung lobe; PP, papillary process; R, right; RA, right atrium; RB, right bronchus; RCLiL, right caudate liver lobe; RILuL, right inferior lung lobe; RLLiL, right lateral liver lobe; RMLiL, right medial liver lobe; RMLuL, right middle lung lobe; RSLuL, right superior lung lobe; RSCV, right superior caval vein; Sp, spleen; St, stomach.

Micro-CT scans of E18.5 foetuses were analysed for the distinct left/right features of visceral organs, including the lung, liver, stomach, colon and spleen (Fig. 3). As previously described (Thiesse et al., 2010), the right bronchus was detected from its first division, which is more cranial than that of the left bronchus (Fig. 3A). In the mouse, the right lung is divided into four lobes (Thiesse et al., 2010) that are clearly identified in micro-CT scans: the right superior lung lobe (RSLuL), which is more cranial; the right middle lung lobe (RMLuL), which is more dorsal and caudal to the RSLuL; the right inferior lung lobe (RILuL), which is more caudal and ventral; and the post-caval lung lobe (PCLuL), which is smaller and located more medially, dorsal to the heart. By contrast, the left lung is composed of a single, large lobe: the left lung lobe (LLuL) (Fig. 3A,E). The mouse liver is also a bilateral and asymmetric organ, located in the abdominal cavity abutting the diaphragm. All liver lobes (Fiebig et al., 2012) can be identified in micro-CT scans (Fig. 3B,C,E). There are two lobes on the left, with the left medial liver lobe (LMLiL), which is more cranial and smaller than the left lateral liver lobe (LLLiL), lying over the stomach. The right liver is subdivided into four lobes. The right medial liver lobe (RMLiL) is cranial and on the right of the gall bladder, whereas the right lateral medial liver lobe (RLLiL) is under the RMLiL and smaller. The right caudate liver lobe (RCLiL) is more caudal abutting the right kidney. Finally, the papillary process (PP) is located between the stomach and the RLLiL, wrapping around the oesophagus. Despite its medial location, clonal analyses indicate that the PP is embryologically more closely related to the right lobes and thus can be considered as a right structure (Weiss et al., 2016). Other visceral organs with an asymmetric position and shape were detected in micro-CT scans (Fig. 3D,E). As in humans, the stomach is located on the left under the LLLiL and the spleen runs along it caudally. The mouse colon, as reported previously (Nguyen et al., 2015; Freeling and Rezvani, 2016), has a single flexure between the proximal and midsegments, hence taking a C-shape, going dorsally and on the left. Thus, micro-CT imaging is a simple and powerful technique to resolve the laterality features of visceral organs, both qualitatively (organ position, asymmetric shape) and quantitatively (organ size, number of lobes). Here, the left and right nomenclature is mainly based on the position within the thoracic or abdominal cavity, with the exception of the PCLuL (connected with the right bronchus) and the PP (which is clonally related to the right liver lobes). We provide annotated 3D reconstructions of organ shape in their endogenous configuration within the body (Movie 3), which will be useful to phenotype mouse models of laterality defects.

### Imaging the position of the heart and its connections with the great vessels by micro-CT

**Fig. 4.**
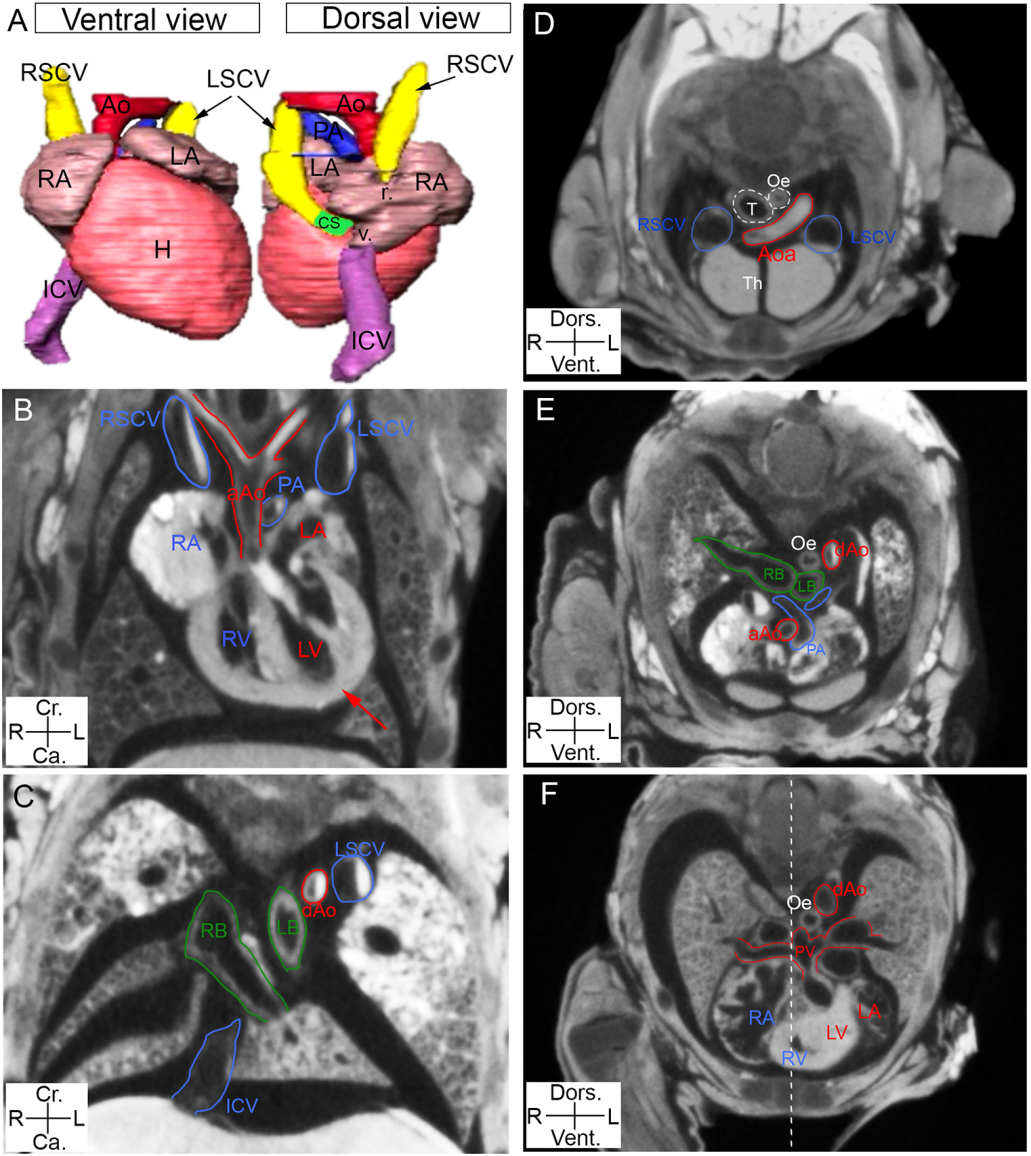
**Imaging of the position of the heart and the great vessels by micro-CT.** (A) Ventral and dorsal views of a 3D reconstruction of the heart (H) and great vessels, after image segmentation. Coronal (B,C) and transverse (D-F, in a cranio-caudal order) sections from micro-CT scans of E18.5 foetuses, showing the position of the heart and the great vessels. The red arrow points to the heart apex, which is on the left (levocardia) in a wild-type sample. The white dotted line represents the plane of bilateral symmetry, bisecting the neural tube. Structures specific to the circulation of deoxygenated and oxygenated blood are annotated in blue and red, respectively; bronchi are in green, other structures in white. aAo, ascending aorta; Aoa, aortic arch; CS, coronary sinus; dAo, descending aorta; ICV, inferior caval vein; LA, left atrium; LB, left bronchus; LV, left ventricle; LSCV, left superior caval vein; Oe, oesophagus; PA, pulmonary artery; PV, pulmonary veins; r., roof of the RA; RA, right atrium; RB, right bronchus; RSCV, right superior caval vein; RV, right ventricle; T, trachea; Th, thymus; v., vestibule of the RA.

As for other visceral organs, the position of the heart within the thoracic cavity is detectable in micro-CT scans based on the location of the apex, which normally points towards the left, a situation referred to as levocardia (Fig. 4A,B). Micro-CT also provides sufficient resolution to analyse the great vessels, which are asymmetric structures. In healthy patients, cardiovascular structures are named or referred to as left or right (shown in red and blue, respectively, in Fig. 4) on the basis of their contribution to the systemic and pulmonary blood circulations, which are driven by the left and right chambers, respectively. The aorta forms an arch towards the left side of the trachea and oesophagus (Fig. 4A-D) and descends on the left of the thoracic cavity (Fig. 4E,F). The aorta is connected to the left ventricle (Fig. 4B), whereas the pulmonary artery, which branches closer to the heart, is connected to the right ventricle. The four pulmonary veins, from the left and right lung lobes, are fused into a single collector that connects to the left atrium (Fig. 4F). The inferior caval vein runs on the right of the body, from abdominal organs to the vestibule of the right atrium (Fig. 4A,C). In the mouse, the right and left superior caval veins, both containing blood from the systemic circulation, arrive from the right and left of the head or upper limbs, respectively; these veins then connect to different regions of the right atrium, the roof and vestibule, respectively (Fig. 4A,B,D) (see Webb et al., 1996 for nomenclature). Thus, by providing a scan of the entire thoracic and abdominal cavities, micro-CT is able to resolve not only the *situs* of visceral organs, but also the complex relative position and connections of the great vessels, which are important features in laterality defects.

### Imaging fine anatomical asymmetries in the heart by HREM

In the clinic, phenotyping the left-right asymmetry of the heart is based on a segmental approach (Van Praagh, 1972), analysing separately the fine anatomical features of the two atria, the two ventricles and the two great arteries beyond their position within the thoracic cavity. This approach requires a histological resolution higher than that of micro-CT, which is achieved by HREM of the explanted E18.5 heart; HREM provides 3D images with a resolution below 4 µm. Such 3D images can be exploited to generate 3D projections of the heart, as well as providing histological sections in any relevant orientation to identify distinct left-right features (Movie 4).

**Fig. 5.**
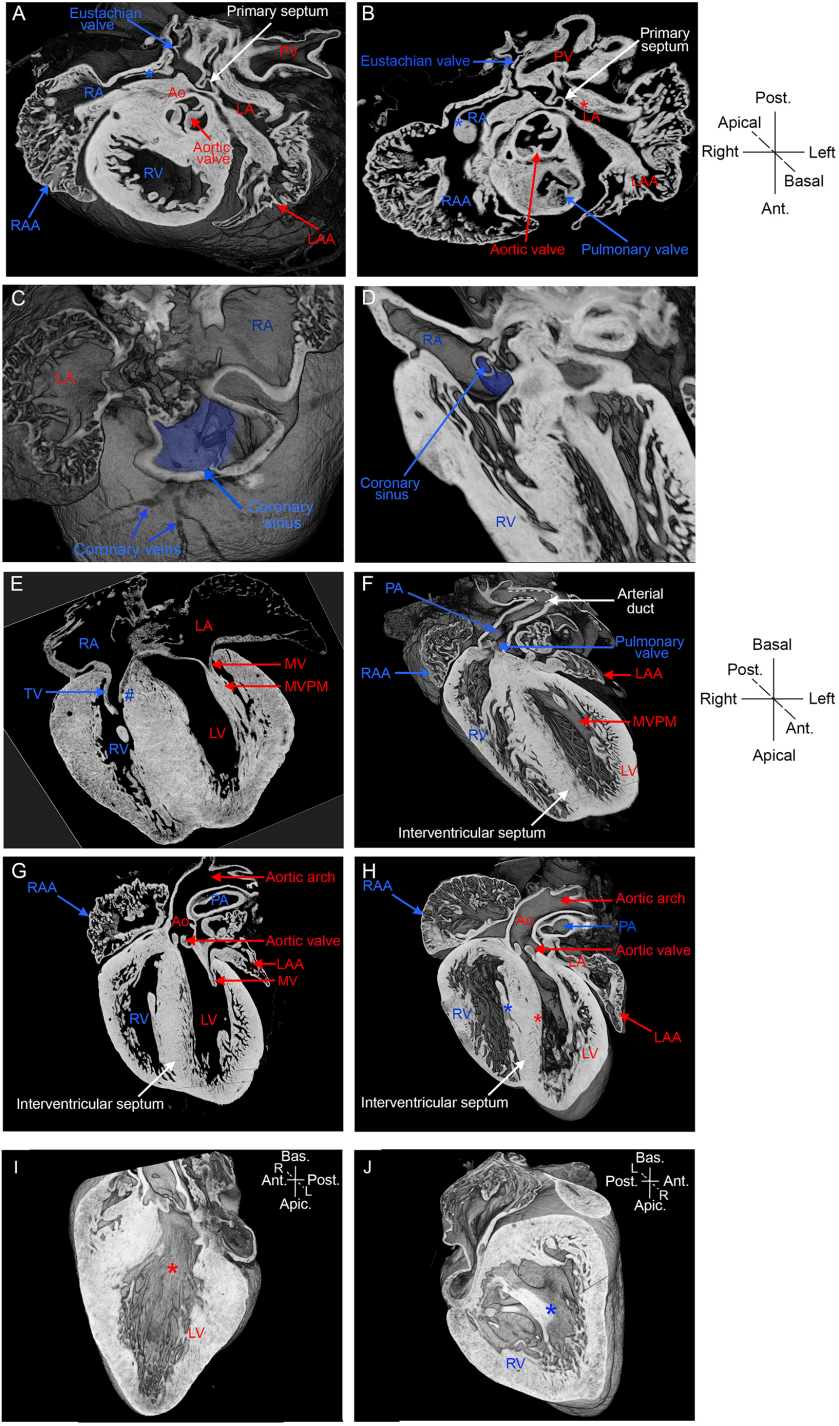
**Imaging the fine cardiac anatomy by HREM.** (A) A 3D projection and (B) transverse section of E18.5 hearts, imaged by HREM, at the level of the arterial valves. The images show the relative positions of the aorta and pulmonary artery, as well as the differential distribution of pectinate muscles in the left and right atrial chambers. The blue asterisk points to a pectinate muscle of the right atrial appendage, which attaches to the Eustachian valve at the end of the inferior caval vein. The red asterisk points to the smooth vestibular region of the left atrium. (C,D) 3D projections showing the coronary sinus, highlighted in blue, collecting the coronary veins and connected to the right atrium. Coronal sections (E,G) and 3D projections (F,H) showing the cardiac chambers, the great arteries and the valves. (I,J) 3D projections of the interventricular septum showing the smooth basal surface in the left ventricle (red asterisk, I) and trabeculated surface in the right ventricle (blue asterisk, J). The tricuspid valve has one septal leaflet (blue #, E). Structures specific to the circulation of deoxygenated and oxygenated blood are annotated in blue and red, respectively; other structures are in white. Apic., apical; Ant., anterior; Ao, aorta; Bas., basal; L, left; LA, left atrium; LAA, left atrial appendage; LV, left ventricle; MV, mitral valve; MVPM, mitral valve papillary muscles; PA, pulmonary artery; Post., posterior; PV, pulmonary veins; R, right; RA, right atrium; RAA, right atrial appendage; RV, right ventricle; TV, tricuspid valve.

The left and right identity of the atria can be distinguished on the basis of their appendages (Uemura et al., 1995). The right atrial appendage has pectinate muscles that extend along the entire atrial chamber, including at the insertion point of the inferior caval vein (asterisk Fig. 5A,B). By contrast, the left atrial appendage corresponds to more confined pectinate muscles at the tip of the atrial chamber (Fig. 5A,B), whereas the vestibular region is smooth. Another asymmetric feature is the specific connection of the right atrium with the inferior caval vein (Van Praagh, 1992) at the level of the Eustachian valve (Fig. 5A,B). The coronary sinus, which in the mouse lies in continuity with the left superior caval vein (Fig. 4A), receives blood from the coronary veins and opens into the right atrium (Fig. 5C,D). The correct configuration, with a morphologically left atrium on the left of the thoracic cavity and the morphologically right atrium connected to the inferior caval vein on the right, is described as atrial *situs solitus* (S).

The right and left ventricles can be identified on the basis of their atrioventricular valves and the trabeculation of the interventricular septum, at a basal level. At the entrance of the right ventricle, the tricuspid valve is more apical compared with the mitral valve of the left ventricle (Fig. 5E). The tricuspid valve has septal attachments, in addition to papillary muscles, whereas the mitral valve has papillary muscles and no septal attachments (Fig. 5E-G). The left ventricle has a smooth septal surface (Fig. 5H,I), whereas the right ventricle has a trabeculated septal surface (Fig. 5H,J). The correct configuration, with the morphologically left ventricle on the left of the thoracic cavity and the morphologically right ventricle on the right, is referred to as a D-loop (D).

In keeping with the rightward rotation of the outflow tract during development (Bajolle et al., 2006; Le Garrec et al., 2017), the position of the great arteries is also a manifestation of left-right asymmetry. The aorta, defined from its connection to the head, normally arises from the left ventricle (Fig. 5G,H) and crosses the pulmonary artery (defined from its connection to the lung), which normally arises from the right ventricle (Fig. 5F). The aortic arch is oriented towards the left of the body (Fig. 5G,H). In transverse sections of the heart, at the level of the aortic and pulmonary valves, the aorta is positioned dorsally and on the right compared with the pulmonary artery (Fig. 5B). In the foetus, the aorta is connected with the pulmonary artery via the arterial duct (Fig. 5F), which will regress after birth. This correct configuration of the great arteries, in the segmental analysis of the heart structure, is referred to as *situs solitus* (S).

With the segmental approach, the nomenclature of a well-formed heart is thus {S,D,S}, in reference to the position of the atria (*situs solitus*), the ventricles (D-loop) and the great arteries (*situs solitus*), respectively. This clinical nomenclature can be applied to the mouse. Another descriptor of the correct alignment of cardiac chambers is atrioventricular concordance, when the morphological right atrium connects with the right ventricle, and vice versa for the left.

In conclusion, HREM provides a high-resolution 3D image of the mouse heart, which is relevant to the phenotyping of the anatomical left/right differences of cardiac chambers. Together with ultrasound imaging and micro-CT, HREM provides an extensive phenotyping of the *situs* and asymmetry of visceral organs, including the cardiovascular system.

### Application of the imaging pipeline to phenotype heterotaxy in *Rpgrip1l* mutants

**Fig. 6.**
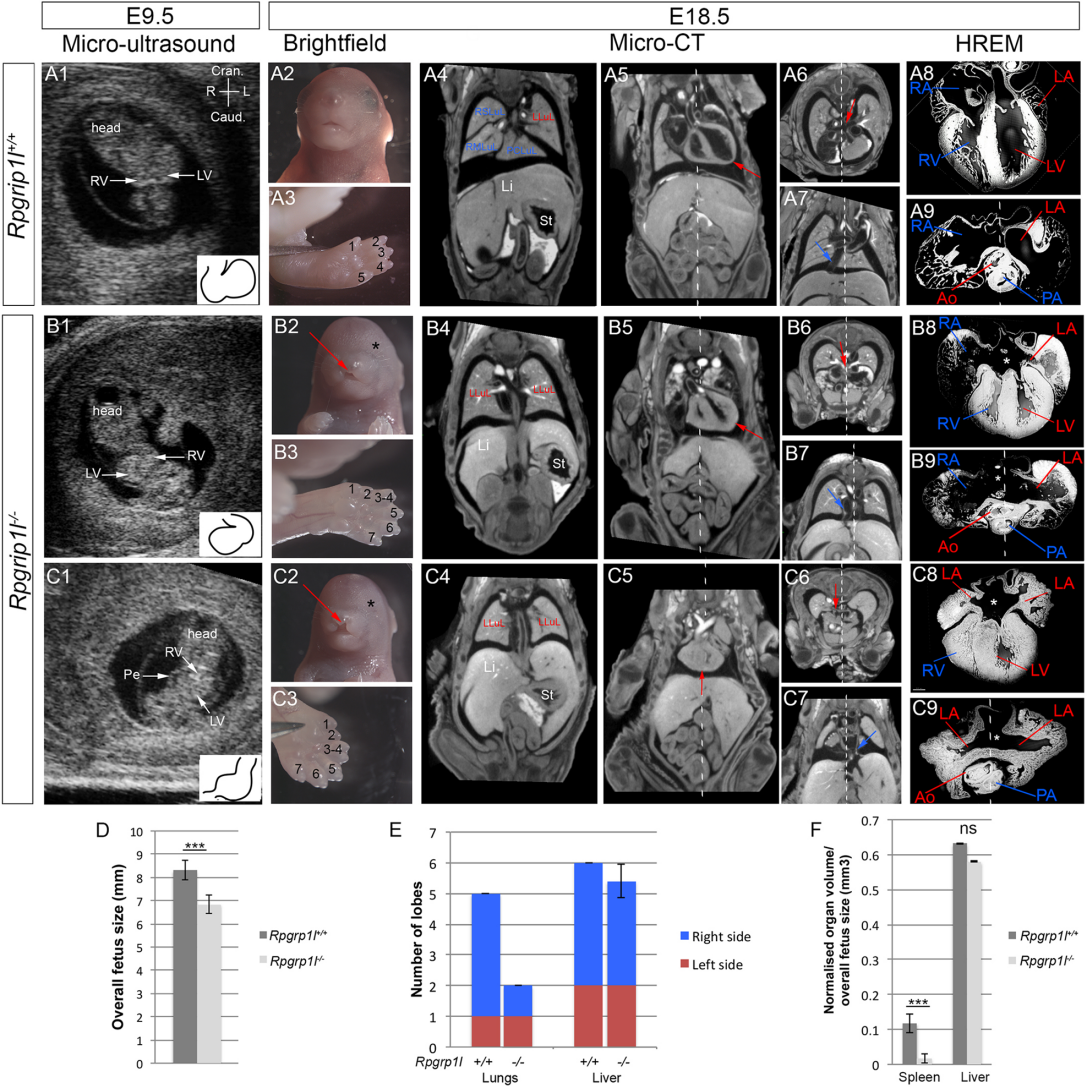
**Application of the pipeline to phenotype heterotaxy: example of *Rpgrip1l* mutants.** (A1,B1,C1) Representative sections of the 3D+t data set from micro-ultrasound imaging of *Rpgrip1l^+/+^*(A1) and *Rpgrip1l^−/−^* (B1,C1) embryos at E9.5. The insets outline the rightward loop of the embryonic heart tube in the control (A1), a reversed loop (B1) or no clear loop direction (C1) in the mutant samples. (A2,B2,C2) Brightfield images of the head of the same individuals at E18.5. The red arrows point to cleft upper lips and the black asterisks to undetectable eyes in mutant foetuses. (A3,B3,C3) Brightfield images of the hindlimb at E18.5, with numbered digits, showing polydactyly in the mutants. Coronal (A4,B4,C4,A5,B5,C5) and transverse (A6,B6,C6,A7,B7,C7) sections of micro-CT scans, showing the lung and stomach *situs* (A4,B4,C4), the position of the heart apex in the thoracic cavity (red arrow in A5,B5,C5), the position of the pulmonary venous return (red arrow in A6,B6,C6) and the position of the inferior caval vein (blue arrow in A7,B7,C7). Left and right anatomical features are annotated in blue and red, respectively. (A8,B8,C8,A9,B9,C9) HREM images of the heart at E18.5 isolated from the same individuals. In coronal sections of the four cardiac chambers (A8,B8,C8), the white asterisk shows complete atrioventricular septal defect in the mutants. In transverse sections (A9,B9,C9), the relative positions of the aorta and pulmonary artery appear normal in these mutants. The white dotted line represents the plane of bilateral symmetry, bisecting the neural tube or the atria. (D-F) Quantitative analyses of micro-CT scans, including the overall body size (D), the lung and liver lobation (E) and the normalised volume of the spleen and liver (F) in control (*n*=8) and mutant (*n*=5) foetuses. Data are presented as means±s.d. ***P<0.01 (Student's t-test). Ao, aorta; Caud., caudal; Cran., cranial; L, left; LA, left atrium; Li, liver; LLuL, left lung lobe; LV, left ventricle; ns, not significant; PA, pulmonary artery; PCLuL, post-caval lung lobe; Pe, pericardial effusion; R, right; RA, right atrium; RMLuL, right middle lung lobe; RSLuL, right superior lung lobe; RV, right ventricle; St, stomach.

We applied our novel imaging pipeline to a previously characterised mouse model of heterotaxy syndrome with a variable penetrance (Delous et al., 2007; Vierkotten et al., 2007). *Rpgrip1l* encodes a ciliary protein localised to the basal body of cilia. It is required for ciliogenesis, a key aspect of the left-right organiser. We performed micro-ultrasound imaging of five independent litters, corresponding to a total of 45 embryos at E9.5 (Fig. S1). In 24/45 embryos, we observed the characteristic shape of the embryonic heart loop (Fig. 6A1). By contrast, 9/45 embryos showed abnormal embryonic heart shapes (Fig. S1A, Table 1), including reversed looping (Fig. 6B1, Movie 5) or a straight heart tube with pericardial effusion (Fig. 6C1). We found that 8/45 embryos were smaller (not distinguishable from delayed E8.5-like embryos) and 4/45 were degenerated or with no heartbeat (Fig. S1). This is consistent with previous observations that showed anomalies of heart looping in *Rpgrip1l* mutants in association with bilateral expression of *Pitx2*, a target of the left determinant Nodal (Vierkotten et al., 2007). As expected from the lethality of *Rpgrip1l^−/−^* mutants *in utero* (Vierkotten et al., 2007), mutant foetuses were not recovered with a Mendelian ratio at E18.5: only half of the expected homozygotes were recovered (Fig. S1C). At E18.5, the 10/45 degenerated decidua and dead foetuses were found in the position of embryos with abnormal or delayed development. Genotyping by PCR in surviving individuals at E18.5 confirmed the homozygous mutation in five foetuses, which had abnormal embryonic heart shape or delayed development at E9.5; the wild-type or heterozygous genotype was identified in the 24 samples that had a normal embryonic heart shape (Fig. S1).

**Table 1.**
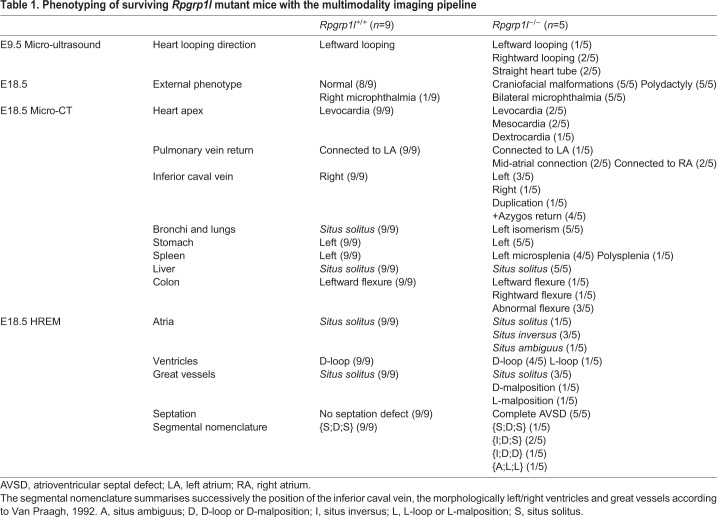
**Phenotyping of surviving *Rpgrp1l* mutant mice with the multimodality imaging pipeline**

The external examination of E18.5 foetuses showed craniofacial malformations, microphthalmia (Fig. 6A2,B2,C2) and polydactyly (Fig. 6A3,B3,C3) in 5/5 alive mutants (Table 1), consistent with previous observations (Vierkotten et al., 2007). By micro-CT, we assessed the overall size of the foetuses and found a reduction in size of the mutant samples (Fig. 6D). Analysis of laterality defects in visceral organs indicated left isomerism of the lungs (Fig. 6A4,B4,C4,E) and bronchi in all mutants (Table 1). Abdominal organ laterality was less affected: only the colon had an abnormal flexure in 4/5 mutants (Table 1). Two liver lobes, the RCLiL and the PP, were reduced or absent in two mutants (Fig. 6E). We quantified organ volume, which was normal for the mutant liver but drastically reduced for the mutant spleen compared with controls (Fig. 6F, Movie 5), corresponding to microsplenia or polysplenia (Table 1). Congenital heart defects were evaluated in micro-CT and HREM images. All mutants showed complete atrioventricular septal defects (Fig. 6A8,B8,C8, Table 1), which precludes the use of valves as a criterion to identify the left and right ventricles. Two mutant foetuses had a normal position of the heart apex, whereas the others showed mesocardia or dextrocardia (Fig. 6A5,B5,C5, Table 1). In 4/5 mutants, the inferior caval vein appeared interrupted at the level of the liver with azygos continuation in the right superior caval vein (Movie 5, Table 1). The inferior caval vein ran normally on the right of the abdominal cavity in one mutant (Fig. 6A7,B7, *situs solitus*), but ran abnormally on the left in three mutants (Fig. 6C7, *situs inversus*); this vein was duplicated in one mutant (*situs ambiguus*). Left isomerism of the atrial appendages was apparent in 3/5 mutant foetuses (Fig. 6A9,B9,C9, Table 1), whereas one had right isomerism and one was normal. A normal ventricular anatomy (D-loop) was identified in 4/5 mutant foetuses, but was associated with an abnormal connection of the pulmonary vein to the right atrium or to the middle of the common atrium (Fig. 6A6,B6,C6, Table 1). Another mutant showed an inverted position of the two ventricles (L-loop) with the pulmonary vein return normally inserted into the left atrium (Table 1). A normal position of the great vessels (*situs solitus*) was identified in 3/5 mutant foetuses (Fig. 6A9,B9,B9, Table 1), 1/5 showed an aorta abnormally positioned in front of the pulmonary artery (D-malposition) and 1/5 had an aorta abnormally positioned on the left of the pulmonary artery (L-malposition). Thus, following the segmental approach (Van Praagh, 1992) and taking into account the combined positions of the inferior caval vein, the morphologically left/right ventricles and the great vessels, *Rpgrip1l^−/−^* mutants can be classified as {S,D,S}, {I,D,S}, {I,D,D} or {A,L,L}, showing variability of congenital heart defects.

By presenting isomerism of the airways, together with atrial and venous return anomalies, the phenotype of *Rpgrip1l* mutants at E18.5 is heterotaxy, according to the criteria of Lin et al. (2014). Using this mutant line, in which an abnormal embryonic heart shape correlates with congenital heart defects and isomerism of the airways, we validate the multimodality imaging pipeline to phenotype laterality defects in the mouse. The extensive phenotyping provided by the pipeline resulted in the identification of novel defects in *Rpgrip1l* mutants, such as isomerism of the bronchi, malrotation of the colon and microsplenia, as well as a range of structural heart malformations, together with the quantification of the penetrance of each defect. The complementarity of the three imaging methods opens the possibility to correlate early embryonic defects with the severity of the heterotaxy phenotype in terms of complex congenital heart defects and the number of visceral organs involved.

## DISCUSSION

On the basis of sequential 3D imaging, the multimodality imaging pipeline that we have developed provides an extensive analysis of left/right anomalies, as well as congenital heart defects, using the nomenclature of paediatric cardiologists in the mouse. We provide annotated reference 3D images of asymmetric organ structures, including the embryonic heart loop at E9.5, visceral organs and great vessels at E18.5. The pipeline combines *in utero* analysis of embryonic shapes, by non-invasive micro-ultrasound imaging, with determination of the *situs* of visceral organs around birth, in their endogenous environment, by micro-CT. Higher-resolution assessment of congenital heart defects is provided by HREM. This standardised pipeline, which was validated in a mouse model of heterotaxy, is essential to phenotype mouse mutants with variable penetrance at multiple scales and multiple stages.

### Multimodality imaging

Previous multimodality imaging approaches have been instrumental in combining functional and anatomical data; for example, *in vivo* Doppler ultrasound imaging combined with micro-CT has been used to assess blood flow distribution in mouse foetuses (Zhou et al., 2014). Standardised phenotyping of mouse mutants at high throughput has been reported based on several high-resolution 3D imaging modalities, including OPT, micro-CT (Dickinson et al., 2016) and HREM (Weninger et al., 2014), together with automated volumetric analyses of organs (Wong et al., 2014). This approach was applied post-mortem at different developmental stages, so that a given individual was imaged once. These consortium screening efforts identified novel mouse mutant lines with lethal phenotypes and incomplete penetrance. Li and colleagues (2015) have performed a wide genetic screen of congenital heart defects, combining ultrasound imaging of the fetal heart (E13.5-E15.5), immediately followed by post-mortem episcopic confocal microscopy of the heart, and determination of visceral organ *situs* by necropsy. Phenotyping was thus at multiple scales, but at a single stage per individual. Heterotaxy is easy to diagnose, however, the origin of the extensive phenotypic variability remains poorly understood. Our approach, which can phenotype several litters in parallel within a month, reaches a similar throughput per mouse line with emphasis on laterality defects. The 3D images of an individual are generated at multiple scales and at multiple stages, as soon as organogenesis in the embryo is initiated. By combining three imaging modalities that are complementary to each other, we now provide a novel framework for describing and correlating different aspects of laterality defects in a single individual.

### Limitations and future extension of imaging modalities

Imaging organogenesis in the mouse embryo in a non-invasive manner is a challenging issue. Here, we focused on the embryonic heart tube, which has been thoroughly quantified in fixed dissected samples (Le Garrec et al., 2017). With high-frequency micro-ultrasound imaging, in *Rpgrip1l* mutants we were able to assess the overall shape of the heart loop of individual embryos through the pregnant mother and to correlate abnormal looping of the embryonic heart with congenital heart defects. It will be possible to extend the analysis to other organs and other stages, without any detected adverse effects on *in utero* development. Micro-ultrasound imaging has a lower resolution than OCT (30-100 µm versus 2-10 µm) (Lopez et al., 2015; Syed et al., 2011), but has a higher imaging depth so it can be used at earlier stages when the deciduum is thicker (E9.5 versus E12.5). Micro-ultrasound imaging is also much less invasive compared with the required externalisation of the uterine horn for OCT. Micro-ultrasound imaging of pregnant mice is amenable to litter sizes of about 10 individuals, beyond which the tracking of each individual in folded uterine horns becomes challenging. With technical development of the probe performance, the resolution of micro-ultrasound imaging is expected to further improve. Emerging methods such as photo-acoustic microscopy, in which acoustic detection can reach a high imaging depth with a resolution varying in depth from 10 to 150 µm, are promising alternatives for *in utero* imaging; however, these techniques are limited to the cardiovascular system, because of the stronger optical absorption of haemoglobin (Laufer et al., 2012).

Given its fast speed of imaging and high resolution, micro-CT has been developed to perform non-destructive imaging of internal organs in mouse mutants (Degenhardt et al., 2010; Dickinson et al., 2016; Wong et al., 2014). Lugol can be used as a relevant contrast agent to visualise soft tissues, such as the heart and vasculature, liver, lung and intestines (Degenhardt et al., 2010); phosphotungstic acid (PTA), which requires a longer incubation but provides higher resolution, can also be used for this purpose (Dullin et al., 2017). In addition to qualitative descriptions of anatomical features, this technique is relevant to morphometric quantifications, as also shown here. Micro-CT has the advantage of being faster and less expensive than magnetic resonance imaging (MRI) (Degenhardt et al., 2010; Norris et al., 2013) and has a wider field of view than OPT (Hsu et al., 2016; Sharpe et al., 2002). A limitation of this technique comes from adverse effects of the contrast agent (Lugol), which induces slight tissue swelling (Degenhardt et al., 2010) and artefact aggregates during pretreatments for HREM. This limitation, which potentially affects image segmentation, did not interfere with the resolution of the asymmetric shape and position of internal organs.

HREM offers an unprecedented histological resolution with 3D rendering to resolve fine anatomical left/right variations. This technique, which is destructive, is used as a final step in the pipeline. It is relevant to morphometric analyses, as well as to quantification of trabeculation or myofibre orientation (Garcia-Canadilla et al., 2018; Paun et al., 2018). HREM was used here for the heart, but could be extended to other explanted organs or the whole foetus (Geyer et al., 2017).

### Differences in organ asymmetry between the mouse and human

Our annotated 3D images and organ segmentations provide a comprehensive reference of asymmetric organs in the mouse. Left-right features in the mouse are generally similar to those of the human with some exceptions (Table 2). The bronchus anatomy is the same with an earlier subdivision of the right bronchus compared with the left, whereas the number of lung lobes is different: four on the right in the mouse compared with three in the human, and one on the left compared with two in the human. In the right lung, the post-caval lung lobe is specific to the mouse (Thiesse et al., 2010). Another lobulated organ in the mouse is the liver, with six lobes that are clearly distinct entities, except for the medial liver, which is continuous between the left (LMLiL) and right (RMLiL) (Fiebig et al., 2012). By contrast, the human liver is a single continuous structure internally, although it is described for surgical purposes as eight segments following surface features (Couinaud, 1957). On either side of the falciform ligament, at the midline, liver segments are grouped in the left and right liver lobes, the right being much larger. In the mouse, as in the human, the stomach and spleen are localised on the left side of the body. In the human colon, the ascending, transverse and descending segments are separated by two flexures (the right/hepatic flexure and the left/splenic flexure), thus taking an inverted U-shape. In the mouse, a single flexure results in a short C-shaped colon (Nguyen et al., 2015; Freeling and Rezvani, 2016). The asymmetry in the shape of the colon is the result of the rotation of the midgut in the embryo (Danowitz and Solounias, 2016).

**Table 2.**
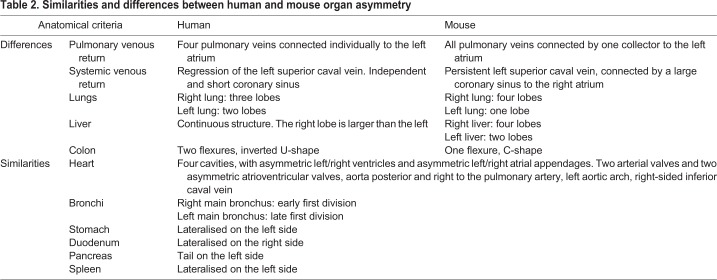
**Similarities and differences between human and mouse organ asymmetry**

The heart anatomy of the mouse is very close to that of the human. Differences are most obvious in the venous return (Webb et al., 1996). The left superior caval vein is persistent in the mouse, whereas it regresses in healthy humans. The coronary sinus remains therefore in continuity with the left superior caval vein in the mouse, whereas its left extremity is closed in the human (Kaufman and Richardson, 2005). Thus, bilateral superior caval veins cannot be considered as a heterotaxy feature in the mouse. In the human, each pulmonary vein is connected individually to the left atrium, whereas in the mouse there is a collector draining together the four pulmonary veins through a single orifice into the left atrium (Webb et al., 1996). The external shape of the atrial appendages is more asymmetric in the human, with a narrow finger-like left atrial appendage and a broad and blunt right atrial appendage (Jacobs et al., 2007). The major determinant of the morphological right atrium in the mouse is the extension of the pectinate muscles to the vestibular region, whereas this region is smooth in the morphological left atrium where the pectinate muscles are confined within the left atrial appendage. The apical trabeculations of the ventricles do not enable differentiation of the right and left chambers in the mouse. All other left-right features are similar in the human and mouse. The aorta, which is posterior and right compared with the pulmonary artery, is connected to the left ventricle; the pulmonary artery is connected to the right ventricle. These great vessels spiral around each other. The aortic arch is on the left side of the body, whereas the inferior caval vein is on the right side. The mitral and tricuspid valves are attached to the atrioventricular junction at different levels along the apicobasal axis (Webb et al., 1996).

### Physiological, anatomical or embryological nomenclature of left/right features

When phenotyping laterality defects, an important question is the rationale used to name asymmetric organ segments as left or right. A physiological perspective predominates in clinical cardiology to distinguish, in reference to healthy patients, the right heart (which drives the circulation of deoxygenated blood) from the left heart (which drives the circulation of oxygenated blood). In this perspective, the inferior and superior caval veins, right atrium and ventricle, and pulmonary artery are defined as right features (shown in blue in Figs 4–5), compared to the pulmonary veins, left atrium and ventricle, and aorta defined as left features (shown in red in Figs 4–5). In some instances, this perspective differs from the anatomical location. The coronary sinus, which drains deoxygenated blood, is considered physiologically as a right structure, although it is connected to the left caval vein in the mouse. The aortic valve, which drains deoxygenated blood, is a physiologically left structure, although it lies on the right of the pulmonary trunk.

From an embryological perspective, the rationale is focused on the origin of cardiac segments, from the pool of left/right precursor cells in the lateral plate mesoderm after gastrulation, which receive distinct molecular signatures, such as Nodal signalling or Pitx2 transcriptional modulation, on the left side (Collignon et al., 1996; Furtado et al., 2011; Liu et al., 2002). The fate and lineage of left versus right heart precursor cells was traced with DiI labelling (Domínguez et al., 2012) and clonal analyses (Lescroart et al., 2010, 2012). From the embryological perspective, the right superior caval vein, the right atrium and the aorta (which derive from right progenitors) are considered as right structures, whereas the left superior caval vein, left atrium, pulmonary veins and pulmonary trunk (which derive from left progenitors) are considered as left structures (see Meilhac and Buckingham, 2018). Thus, for the aorta, pulmonary trunk and left superior caval vein, there are discrepancies between the physiological and embryological perspectives. This might also affect the coronary sinus, which lies in continuity with the left superior caval vein. The left atrium was shown to receive a contribution from both right and left precursors (Domínguez et al., 2012). The origin of the ventricles has not yet been demonstrated, although the process of fusion of the heart tube would suggest a double left/right origin for each ventricle; this idea is further supported by genetic tracing with the left determinant Pitx2 (Ai et al., 2006; Furtado et al., 2011; Liu et al., 2002). The embryological perspective thus differs from the anatomical location in the case of cardiac chambers. This is because the anatomical location of cardiac segments is the result of asymmetric morphogenesis, i.e. the rightward looping of the heart tube (Le Garrec et al., 2017) or the rightward rotation of the outflow tract (Bajolle et al., 2006).

In summary, our multimodality imaging pipeline offers novel perspectives for an exhaustive phenotyping of mouse mutants and provides new insight into the embryonic origin of laterality defects and the mechanisms of congenital heart malformations. Beyond cardiovascular research, applications of this novel pipeline can easily be extended to the study of asymmetric morphogenesis of other organs. The fast and standardised collection of images is relevant to the new automated and quantitative image analysis procedures that are emerging.

## MATERIALS AND METHODS

### Animal models

Control embryos were from a mixed genetic background. The *Rpgrip1l^+/−^* mouse line (a gift from S. Schneider-Maunoury) was maintained in a C57Bl6J genetic background. Male and female samples were mixed. E0.5 was defined as noon on the day of vaginal plug detection. Animal procedures were approved by the ethical committee of the Institut Pasteur and the French Ministry of Research.

### Micro-ultrasound imaging

Pregnant female mice were anaesthetised with 4% isoflurane (in oxygen) for induction and 2% for maintenance. The abdomen was shaved using a depilatory cream to minimise ultrasound attenuation. The animal was restrained on a heated platform with surgical tape, maintaining a normal body temperature during imaging. Heart rate, temperature and breathing were monitored with paw and rectal probes. Ultrasonic gel was applied on the skin to perform non-invasive transabdominal imaging using the Ultra High-Frequency Imaging Platform Vevo2100 (Visualsonics) with a 50 MHz probe (MS-700). Fast scans of the uterine horns and of each embryo were acquired to identify the embryo position in the uterus. A 3D+t scan of each embryo (E9.5) was acquired across the deciduum. The data set comprises 94 images with an axial and lateral resolution of 30 and 50 µm, respectively. The motor has a step size of 32 µm.

### Micro-CT

Foetuses were recovered before birth, at E18.5, and their position in the uterine horns was carefully monitored. Foetuses were euthanised by decapitation. The thoracic skin was removed to allow penetration of the contrast agent; the left arm was removed, as a landmark of the left side. Blood was washed out in PBS and the heart was arrested in diastole with 250 mM cold KCl. Samples were stained in 100% Lugol (Sigma-Aldrich) over 72 h (Degenhardt et al., 2010). Images of the thorax and abdomen were acquired on a Micro-Computed Tomography Quantum FX (Perkin Elmer), within a field of exposure of 10 mm diameter. The data set, comprising 512 images of 20×20×20 µm xyz resolution for each sample, was analysed using OsiriX Lite software.

### HREM

A control E9.5 embryo was dissected, incubated in cold 250 mM KCl, fixed in 4% paraformaldehyde and washed in PBS. E18.5 hearts were isolated after micro-CT imaging, washed in PBS to remove Lugol staining as much as possible, post-fixed in 4% paraformaldehyde and washed in PBS. Samples were dehydrated in series of methanol concentrations and embedded in methacrylate resin (JB4, Polysciences) containing Eosin and Acridine Orange as contrast agents (Geyer et al., 2017; Weninger et al., 2006; 2014). Single-channel images of the surface of the resin block were acquired using the Optical High-Resolution Episcopic Microscope (Indigo Scientific) and a GFP filter, repeatedly after removal of 1.56 µm (at E9.5) or 2.34 µm (at E18.5) thick sections. The data set comprised 1200 images of 1.45-2.43 µm resolution in x and y at E9.5 and 1600 images of 2.3-3.8 µm resolution in x and y at E18.5.

### Image analysis

Image sequences acquired by micro-ultrasound imaging were post-treated to generate a cubic resolution and volume rendering using the Volume Viewer plugin from Fiji (ImageJ). The volume was resectioned to generate standardised coronal views, independently of the orientation of image acquisition. The 3D images acquired by micro-CT were segmented manually with AMIRA software (Thermo Fisher Scientific) or OsiriX MD (Pixmeo) and volumes were extracted from the regions of interest. The overall size of E18.5 foetuses was measured as the distance from the thymus to the left hip. The 3D images acquired by HREM were analysed using Imaris software (Bitplane): the heart at E9.5 was segmented as described in Le Garrec et al., 2017 and surface rendering of the E18.5 heart and optical sections was performed with the oblique slicer. Volume rendering of the E18.5 heart was performed with the Volume Viewer plugin from Fiji (ImageJ), after adjustment of the resolution as cubic.

### Statistics

The collection of full litters was used to randomise imaging experiments. Group allocation was based on PCR genotyping. Investigators were blinded to allocation during imaging and phenotypic analysis, but not during quantifications. Sample size was checked *post hoc*, using the calculator powerandsamplesize.com, in order to ensure a power of at least 0.8 with a type I error probability of 0.05 and an effect size of 10% (Fig. 6D, liver in 6F) or 50% (spleen in Fig. 6F). All sample numbers indicated in the text refer to biological replicates (i.e. different embryos or foetuses). No outlier was excluded from the data analysis. Data in Fig. 6D and F follow a normal distribution and the variance is not significantly different between controls and mutants (*F*-test). Comparisons of two-centre values were performed on the average, or the geometrical mean when ratios were compared, using a Student's two-tailed test. Tests were performed with Excel.

## Supplementary Material

Supplementary information
