# Supplementary material for: Standardised imaging pipeline for phenotyping mouse laterality defects and associated heart malformations, at multiple scales and multiple stages
Source: Dis Model Mech. 2019 Jul 9;12(7):dmm038356. doi: 10.1242/dmm.038356 (PMC6679386; doi:10.1242/dmm.038356)
Supplement: Supplementary information [file dmm-12-038356-s1.pdf]

Supplementary Information

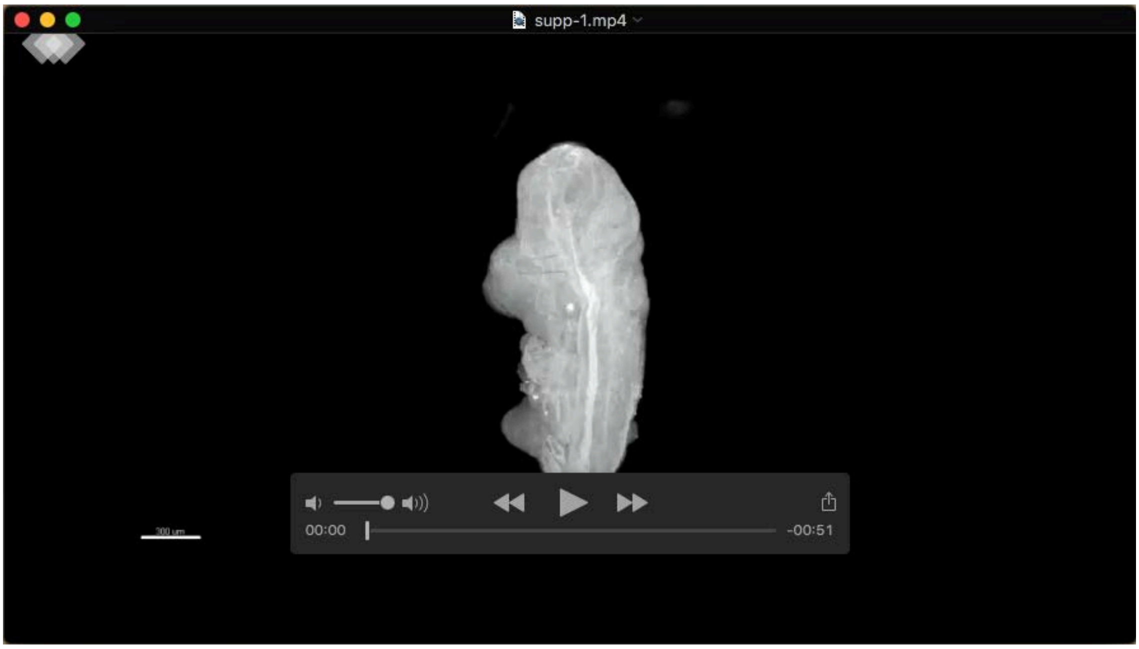

Movie 1: Example of a 3D reconstruction of an embryonic heart at E9.5 by HREM. The first sequence shows a 3D projection, rotating rightward, of an E9.5 embryo imaged by HREM. The second sequence shows serial transverse sections of the thoracic region. The final sequence displays a 3D reconstruction of the embryonic heart shape, after image segmentation. It is first presented within the image of the thoracic region, and then isolated, using the notochord as an axial reference. AVC, atrioventricular canal. x/y resolution,1.45 µm; z resolution,1.56 µm.

Disease Models & Mechanisms • Supplementary information

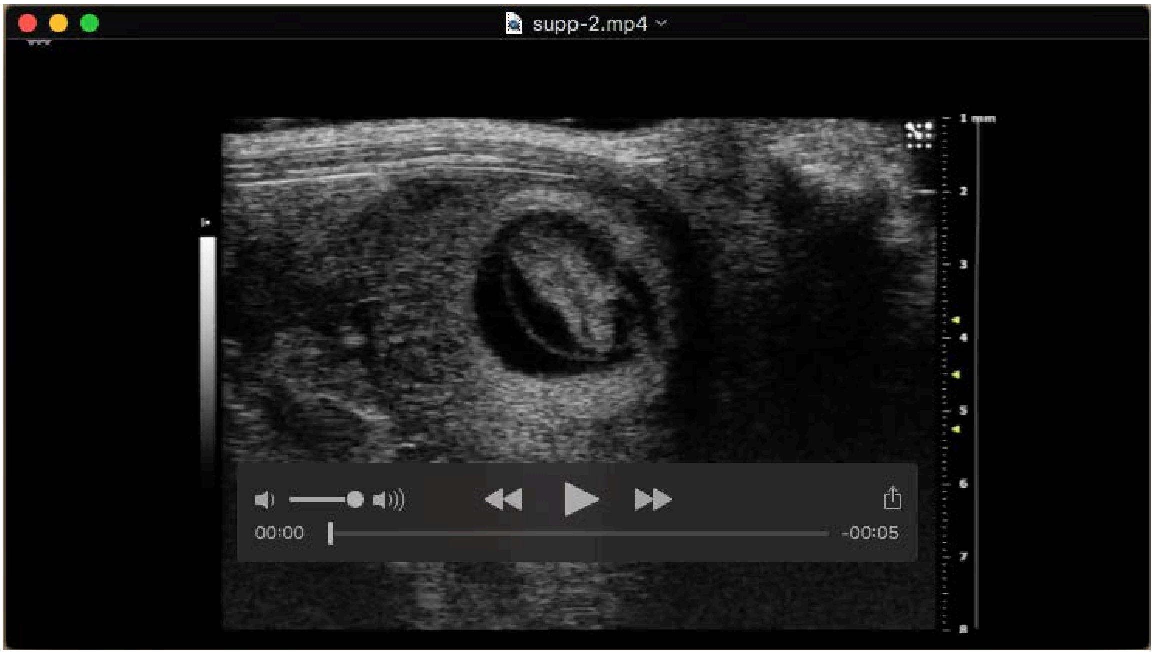

Movie 2: Example of a 3D+t image by micro-ultrasound imaging **at E9.5.** Fast scan of an E9.5 embryo from ventral to dorsal. x/y resolution, 50 µm; speed of acquisition, 100 frames per second.

Disease Models & Mechanisms • Supplementary information

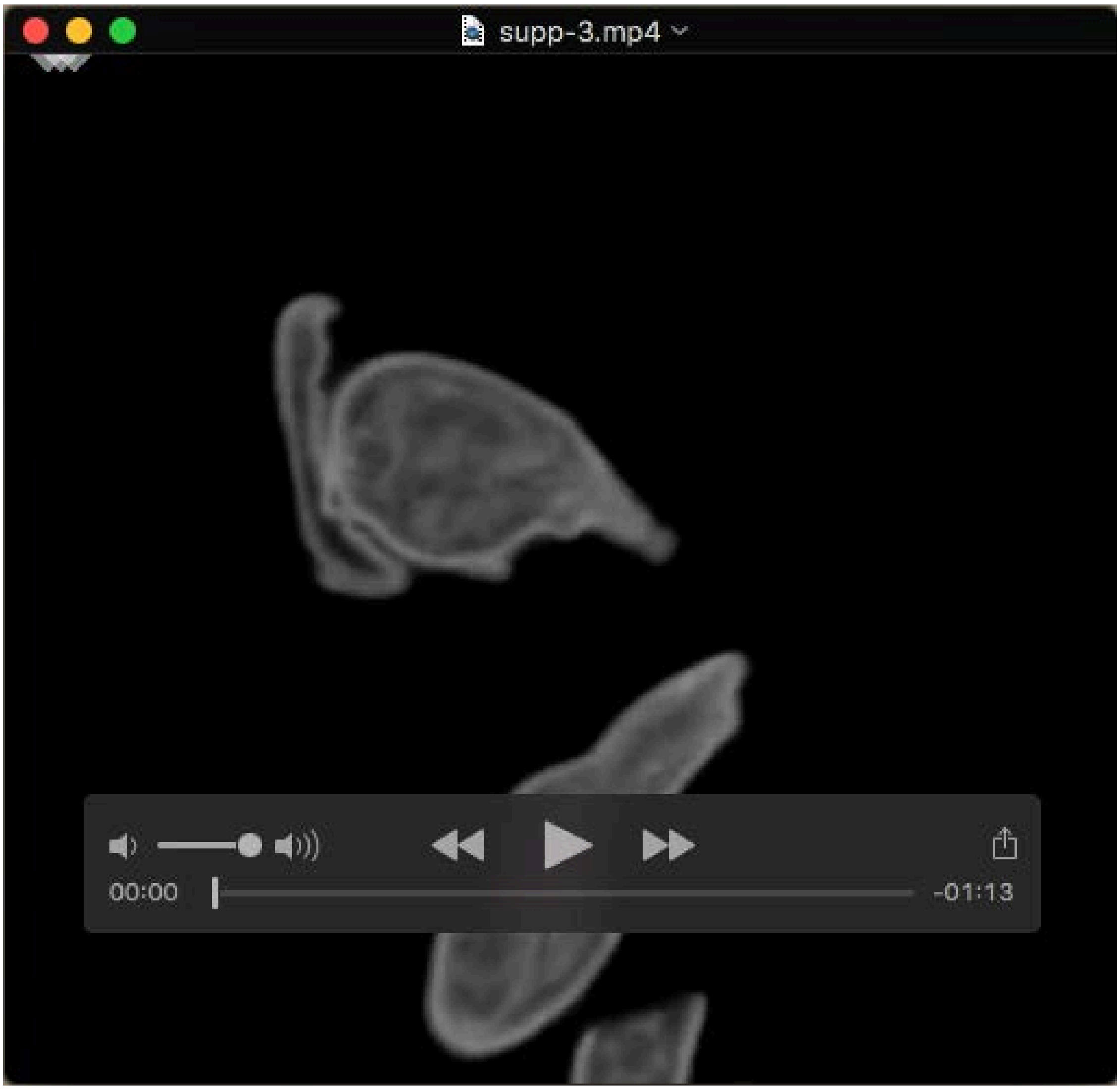

Movie 3: Example of a micro-CT scan and 3D reconstruction of visceral organs at E18.5. The first sequence shows coronal sections of a scan, acquired by micro-CT, of an E18.5 fetus. The second sequence shows the 3D projection of the scan, later with visceral organs highlighted in colours. In the final sequence, 3D reconstructions show the situs of thoracic (lungs and heart) and abdominal (liver, stomach, spleen and colon) organs, as well as the anatomy of bronchi, and the connections of the great vessels to the heart. See Figure 3E and 4A for the colour code and annotations. x/y/z resolution, 20 µm.

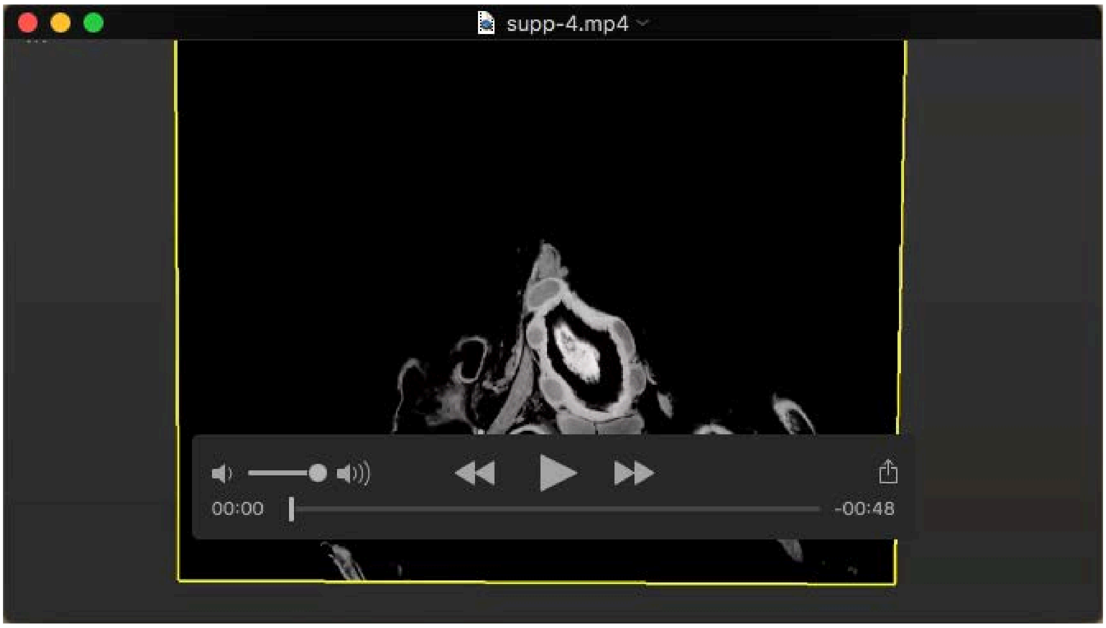

Movie 4: Example of heart imaging by HREM at E18.5. Serial transverse sections and 3D projection are shown sequentially. LAA, left atrial appendage; RAA, right atrial appendage. x/y resolution, 2.9 µm; z resolution, 2.34 µm.

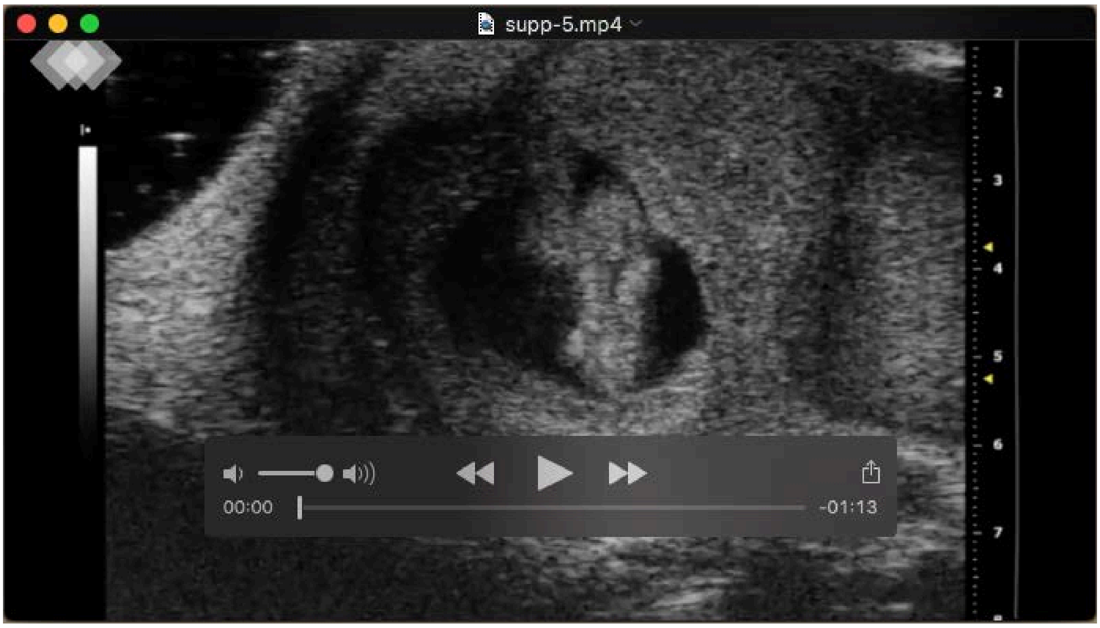

Movie 5: Application of the multimodality imaging pipeline in a *Rpgrip1l*$^{-/-}$ embryo. The first sequence is a fast scan of the *Rpgrip1l*$^{-/-}$ E9.5 embryo shown in figure 6B, from ventral to dorsal. x/y resolution, 50 µm. The second sequence is a micro-CT scan of the same individual at E18.5, showing in parallel coronal (on the left) and transverse (on the right) optical sections. x/y/z resolution, 20 µm. The left isomerism of the lungs, hypoplasia of the pulmonary artery branches (PAB), the azygos return of the right superior caval vein are indicated. The final sequence shows serial transverse sections of the isolated heart at E18.5 acquired by HREM and the corresponding 3D projection. x/y resolution, 2.9 µm; z resolution, 2.34 µm. The atrioventricular septal defect is indicated with an asterisk. Ao, aorta; Co, colon; LA, left atrium; LLuL, left lung lobe; LV, left ventricle; PA, pulmonary artery; RA, right atrium; RV, right ventricle; St, stomach, Sp, spleen.

Disease Models & Mechanisms • Supplementary information

Desgrange et al.
Figure S1

A

Litter	Embryo	Phenotype at E9.5 (micro-ultrasound)	Phenotype at E18.5 (brightfield)	Genotype
#1	R1	Normal	Normal	*Rpgrip1l*$^{+/+}$
	R2	Abnormal looping	Degenerated	NA
	R3	Normal	Normal	*Rpgrip1l*$^{+/+}$
	R4	Smaller	Degenerated	*Rpgrip1l*$^{-/-}$
	L1	Normal	Normal	*Rpgrip1l*$^{+/-}$
	L2	Smaller	Degenerated	NA
	**L3**	**Abnormal looping**	**Cranofacial malformations and Polydactyly**	***Rpgrip1l*$^{-/-}$**
	L4	Normal	Normal	*Rpgrip1l*$^{+/-}$
	L5	No heartbeat	Degenerated	NA
#2	R1	Normal	Normal	*Rpgrip1l*$^{+/+}$
	**R2**	**Abnormal looping**	**Cranofacial malformations and Polydactyly**	***Rpgrip1l*$^{-/-}$**
	R3	Normal	Normal	*Rpgrip1l*$^{+/-}$
	R4	Smaller	Normal	*Rpgrip1l*$^{+/-}$
	R5	Normal	Normal	*Rpgrip1l*$^{+/+}$
	L1	Abnormal looping	Degenerated	NA
	L2	Abnormal looping	Degenerated	NA
	L3	Normal	Normal	*Rpgrip1l*$^{+/+}$
	L4	Normal	Normal	*Rpgrip1l*$^{+/+}$
#3	R1	Normal	Normal	*Rpgrip1l*$^{+/-}$
	R2	Normal	Normal	*Rpgrip1l*$^{+/-}$
	**L1**	**Smaller**	**Cranofacial malformations and Polydactyly**	***Rpgrip1l*$^{-/-}$**
	L2	Normal	Normal	*Rpgrip1l*$^{+/-}$
	L3	Degenerated	NA	NA
	L4	Degenerated	NA	NA
	L5	Normal	Normal	*Rpgrip1l*$^{+/+}$
	L6	Normal	Normal	*Rpgrip1l*$^{+/+}$
#4	R1	Degenerated	NA	NA
	R2	Abnormal looping	Degenerated	NA
	**R3**	**Abnormal looping**	**Cranofacial malformations and Polydactyly**	***Rpgrip1l*$^{-/-}$**
	R4	Normal	Normal	*Rpgrip1l*$^{+/-}$
	R5	Smaller	Degenerated	*Rpgrip1l*$^{+/-}$
	R6	Smaller	Normal	*Rpgrip1l*$^{+/-}$
	L1	Normal	Normal	*Rpgrip1l*$^{+/-}$
	L2	Normal	Normal	*Rpgrip1l*$^{+/+}$
	L3	Normal	Normal	*Rpgrip1l*$^{+/-}$
	L4	Normal	Normal	*Rpgrip1l*$^{+/-}$
#5	R1	Normal	Normal	*Rpgrip1l*$^{+/-}$
	R2	Normal	Normal	*Rpgrip1l*$^{+/-}$
	**R3**	**Abnormal looping**	**Cranofacial malformations Polydactyly**	***Rpgrip1l*$^{-/-}$**
	R4	Normal	Normal	*Rpgrip1l*$^{+/-}$
	R5	Smaller	Normal	*Rpgrip1l*$^{+/+}$
	R6	Smaller	Right microphtalmia	*Rpgrip1l*$^{+/+}$
	L1	Normal	Normal	*Rpgrip1l*$^{+/-}$
	L2	Normal	Normal	*Rpgrip1l*$^{+/-}$
	L3	Abnormal looping	Degenerated	NA

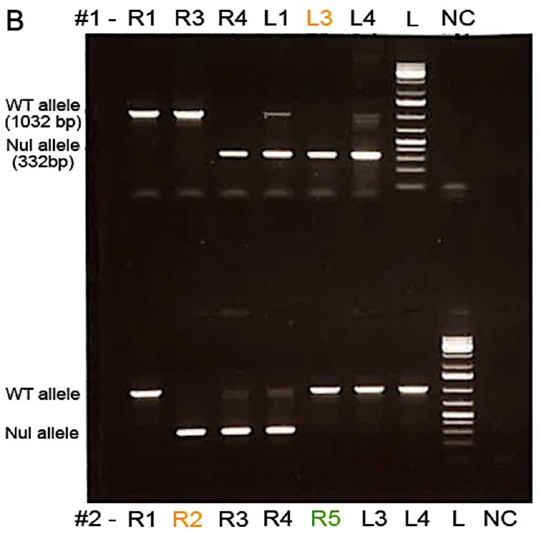

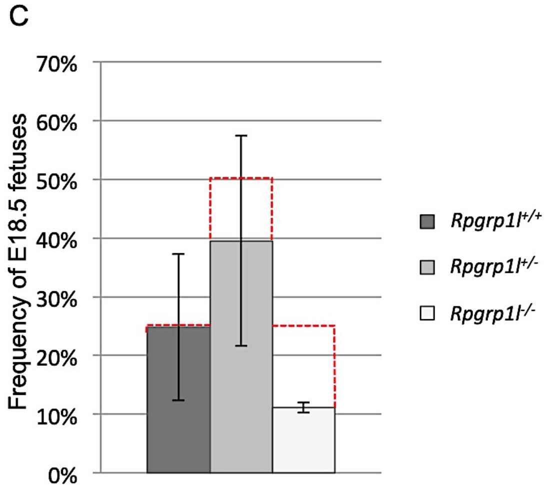

Figure S1. Genotype/phenotype correlation and mendelian ratio in litters from *Rpgrip1l*$^{+/-}$ intercross. (A) Gross phenotype of developing mice, observed by micro-ultrasound imaging at E9.5 and then externally at the time of dissection at E18.5. Individuals are numbered by

Disease Models & Mechanisms • Supplementary information

litter (#1 to #5) and position in the uterus (see Figure 1A). *Rpgrp1l*$^{-/-}$ which survived to E18.5 are indicated in bold. (B) PCR results showing examples of genotyping. (C) Histogram showing the frequency of recovery of the indicated genotypes per litter at E18.5. The red doted lines show the expected mendelian ratio. Data are presented as mean ±standard deviation L, ladder; NA, not applicable; NC, negative control (water). Images of individuals #2R5, #2R2 and #1L3 are shown in Figure 6A, B and C respectively.

Disease Models & Mechanisms • Supplementary information
